# Identification of two novel *OPA1* mutations in Chinese families with autosomal dominant optic atrophy

**Published:** 2008-12-29

**Authors:** Yang Li, Ting Deng, Yi Tong, Shuling Peng, Bing Dong, Dacheng He

**Affiliations:** 1 Beijing Institute of Ophthalmology, Beijing Tongren Hospital, Capital Medical University, Beijing, China; 2 Key Laboratory for Cell Proliferation and Regulation Biology, Ministry of Education, Beijing Normal University, Beijing, China; 3 The First Affiliated Hospital, Fujian Medical University, Fuzhou, Fujian, China

## Abstract

**Purpose:**

To report the clinical features and identification of two novel mutations in two Chinese pedigrees with autosomal dominant optic atrophy (ADOA).

**Methods:**

Two families (F1 and F2) including ten affected members and nine unaffected family individuals were examined clinically. After informed consent was obtained, peripheral blood samples of all the participants were obtained, and genomic DNA was extracted. Linkage analysis was performed with two microsatellite markers around the *OPA1* gene (D3S2305 and D3S3562) in family F1. The coding region (exon 1–28), including intron-exon boundary of the *OPA1* gene, were screened in the 2 families by polymerase chain reaction (PCR) and direct DNA sequencing. Whenever substitutions were identified in a patient, single strand conformation polymorphism (SSCP) analysis was performed on all available family members and 100 normal controls. To characterize a splicing site mutation, RT–PCR of total RNA of leukocytes obtained from three patients and seven unaffected individuals of family F1 was performed with the specific primers.

**Results:**

The affected individuals all presented with bilateral visual failure and temporal or total pallor of the optic discs. Genotyping of family F1 revealed the linkage to the *OPA1* gene on 3q28–29. After sequencing of *OPA1* gene, a novel heterozygous splicing site mutation c.985 −2A>G in intron 9 was found in family F1. RT–PCR result showed the skipping of the exon 10 in the mutant transcript, which results in loss of 27 amino acids in the OPA1 protein. A novel heterozygous nonsense mutation c.2197C>T(p.R733X)was detected in family F2.

**Conclusions:**

Our findings expand the spectrum of *OPA1* mutations and further established the role of *OPA1* gene in Chinese patients with ADOA.

## Introduction

Autosomal dominant optic atrophy (ADOA, OMIM 165500), also called Kjer type optic atrophy, is the most frequent form of inherited optic neuropathy with a prevalence of between 1:10,000 and 1:50,000 [1,2]. It is a disorder characterized by slowly progressive visual loss usually starting in childhood, color vision defects, centrocecal scotomas, and temporal optic nerve pallor [3]. The results of histopathological examinations of affected donor eyes suggest that the fundamental pathology is degeneration of the retinal ganglion cells and loss of myelin and nerve tissue within the optic nerve [4]. ADOA is genetic heterogeneous, and 4 chromosome loci for the ADOA have been mapped [5–8]. Of the 4 loci, only *OPA1* gene (3q28–29) [5] and *OPA3* gene (19q13) [7] have been identified. Mutations in the *OPA1*gene account for the majority of cases of ADOA [9,10], while mutations in the *OPA3* gene cause ADOA with cataract [7].

The *OPA1* gene on chromosome 3q28–29 consists of 28 coding exons and encodes a 960 amino acid polypeptide [9,10]. Due to alternative splicing of exon 4, 4b, 5b, the *OPA1* gene has 8 mRNA splicing isoforms with different expression in different tissues. In human retina and brain, isoform 1 (missing exon 4b and 5b) and isoform 4 (missing exon 4 and 4b) have been shown to have the predominate expression [11]. OPA1 protein is a dynamin-related GTPase targeted to mitochondria, which locates mostly on the mitochondrial inner membrane [12,13]. OPA1 consists of 5 domains: mitochondrial target signal (MTS), N-terminal (N-terminal) coiled-coil domain, GTPase domain, dynamin central region, and C-terminal (C-terminal) coiled-coil domain [13]. OPA1 is thought to be involved in multiple functions, the key role being the regulation of mitochondrial dynamics, the maintenance of structural integrity of the cristae [12–14], and regulation of the apoptotic process through the control of cytochrome C redistribution [12–14].

To date, more than 100 mutations have been reported in the *OPA1* gene and most of them are localized to the N-terminal leader sequence (exon 1–2), the GTPase domain (exon 8–16), and C-terminal coiled-coil region (exon 27–28) [9–11,15–24].

In this study, we performed a mutation screening of the *OPA1* gene in two Chinese families affected with ADOA and identified two novel mutations.

## Methods

### Patients and DNA samples collection

This study was granted approval by the Beijing Tongren Hospital Joint Committee on Clinical Investigation and conformed to the tenets of the Declaration of Helsinki. After informed consent was obtained, each participant (two probands were examined in the Beijing TongRen Hospital, the other participants, including 14 individuals of family 1 and 3 members in family 2, were examined in their local town by Dr. Li and Dr.Tong) underwent clinical examinations including best-corrected visual acuity using E decimal charts, slit-lamp, and ophthalmoscope. Two probands of the families underwent visual field, pattern visual evoked potential (P-VEP), and color discrimination test. ADOA was diagnosed based on the clinical and family history, the bilateral visual loss, and temporal pallor of optic discs. Peripheral blood was obtained by venipuncture in heparinized collecting tube and kept in 4 °C less than one week, and genomic DNA was extracted according to standard phenol protocols.

### Linkage analysis

Genotyping and linkage analysis were performed with two microsatellite markers D3S2305 and D3S3562 around the *OPA1* gene in family F1. The fine mapping primer sequences were obtained from the GDB Human Genome Database. Pedigree and haplotype map were constructed using Cyrillic V. 2.0 software.

### Mutation screening of the *OPA1* gene

Mutation screening was performed in the two families using direct DNA sequence analysis. Primers of 1–28 exons and exon-intron boundaries of the *OPA1* gene were designed by the Primer3 program. Details of the primer sequences are shown in Table 1. For direct sequencing, PCR products were purified (Shenneng Bocai PCR purification kit; Shenneng, Shanghai, China). The purified PCR products were sequenced using an automatic fluorescence DNA sequencer (ABI, Prism 373A; Perkin Elmer, Foster City, CA), according to the manufacturer’s instructions. Nucleotide sequences were compared with the published DNA sequence of *OPA1* isoform 1 (GenBank accession number NM_015560.1) using DNAssit Version 1.0. For *OPA1* gene cDNA numbering +1 corresponds to A in the ATG translation initiation codon of *OPA1* isoform 1.

**Table 1:** PCR primers used in this study

**Primer**	**Forward (5′-3′)**	**Reverse(5′-3′)**
Exon1	CCACTTCCTGGGTCATTCC	AGAATTAACGGGGCCAGATT
Exon 2	CCCTCTCTGATCTTTCTTCCAT	TAATTGGAAAACCAGGAGGA
Exon 3	TATTTGGCATGCAGAGCATC	TCTCTTTCCTCGAGATGACCA
Exon 4	GGGTTGTCATGAGGATTAAACAA	CATGTATTTTTCCTCCATGGTTC
Exon 5	AAAGGCGATTTGATTCTTTGAA	TCTTTCAAGACTACCTACATGAACAA
Exon 6	AAAAATTTAACTTGCTGTACATTCTG	CACCTTCCAAATTTTGCTCTG
Exon 7	TCAAGATTTTGGAAGATTTTAATTTAG	CACACAACGTTAAGCGGTAAAA
Exon 8	CCGTTTTAGTTTTTACGATGAAGA	TTTTTGCTAGTTGGCAAGTTCA
Exon 9	AAAAACTCAGAGCAGCATTACAAA	CCTAAGGAACCTCACTGAGACG
Exon 10,11	CATACGGGCTGTGGGAATTA	CCATAAAACGTCACTGAAATGAA
Exon 12,13	GAATTTTTAGAATACATTTCACCAAAA	TGGATTGCTAAAGAAGAAAACAT
Exon 14	GACACAGGGGTATAATTTGTACTGA	TTCTCGCAACAAAGAATTTGA
Exon 15,16	TTTTGCTTTCTAAATTGTATATTACGC	TGAAAACAGTTCAATTTAAGCTACTC
Exon 17	CATTCGCAGACTTGGTGGTA	TGTCTTAATTTGCTTGCTTCTTT
Exon 18	CCACTTTAACCACTACATCTGGAA	AGCTTATCAGATTTTTCTCTCAACA
Exon 19	TCTGAAAATCATGACAGGGTAAA	CAAGGCAACAATAAATCACTGC
Exon 20	TGATACTTCAGTCAAGCTGTTTTT	CAGCTCCTACTCCCTTCAGA
Exon 21	TTTTTCATGTTAACCATTGAAGTATG	GAGGCTGATACCCCAGTATACAA
Exon 22	TTTTTCCATATTTACTAAGCTGTCAA	TCACCACTGTGAACTCAGAACTC
Exon 23	TTCCTTTATTTCAACTGCCTTCA	AATGCCTGAATTAAAATGAACAA
Exon 24	TCAAGCACCAAATTATGAACCA	GCAGATTCCTGCTTCTCAGC
Exon 25	TGTACAACTTCTCAGTGTGGTTGA	GCATATTTTGACAACTGTTGCTT
Exon 26	AAGCTTAGGACATATCTACTGGTTCT	TGGGAAGTATTTTGGCATCC
Exon 27	TCTTTATTCATTTATAAAAACGATGC	AAATGGGAAAGGTGGAAAGG
Exon 28	CCTCCTGATTTGTGATACCTTTG	CAAGCAGGATGTAAATGAAGCA

### Single strand conformation polymorphism

To confirm the variations found in the sequencing, we conducted single strand conformation polymorphism (SSCP) analysis in all available family members and 100 normal controls. Amplified DNA was mixed with an equal volume formamide buffer that contained 95% formamide, 10 mM EDTA, 0.1% bromophenol blue, and 0.1% xylene cyanol. Denatured samples were electrophoresed on a 16% non-denaturing polyacrylamide gel (acrylamide:bisacrylamide=49:1) in Tris-borate-EDTA buffer (89 mM Tris base, 2 mM EDTA, and 89 mM Boric acid, pH 8.0) for 12–16 h at 300 v and 4 °C before gels were silver-stained and analyzed [25].

### Reverse transcription-polymerase chain reaction

Lymphocytes from peripheral blood were isolated from the 3 patients and 7 unaffected individuals of family F1. Total RNA was extracted with TRIZOL Reagent (Invitrogen, Carlsbad, CA). Reverse transcription of isolated RNA was performed using the First-Strand cDNA Synthesis Kit (Toyobo, Osaka, Japan) with random primers. The transcribed *OPA1* fragment from exon 9 to exon 11 was amplified using the following primer pair: forward, 5′- TGG AGA TCA GAG TGC TGG AA-3′; and reverse, 5′- CTC AGG GCT AAC GGT ACA GC-3′. RT–PCR products were separated on a 1% agarose gel and sequenced.

## Results

### Clinical findings

**Figure 1:**
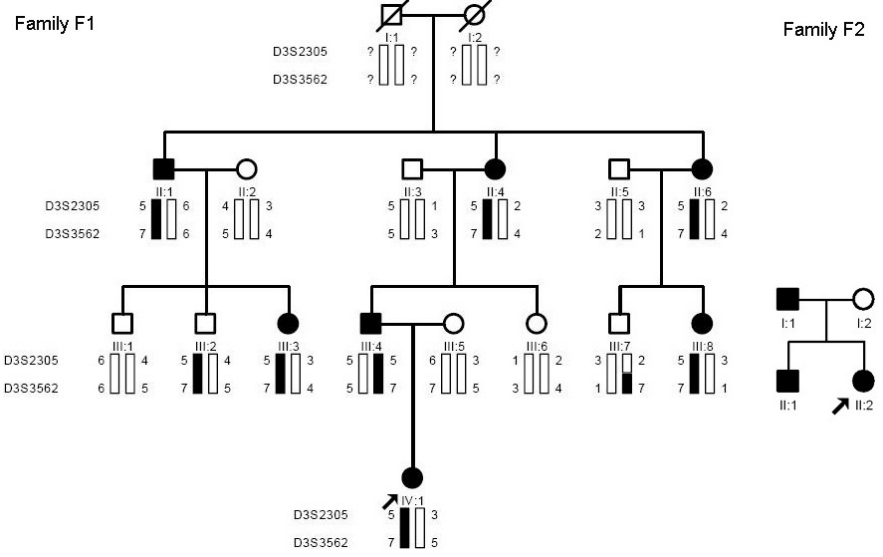
Family structure and haplotype analysis of the two Chinese families with ADOA. Pedigree of the two families with autosomal dominant optic atrophy (ADOA) and haplotype analysis of the family F1 showed segregation two microsatellite markers on chromosome 3 listed in descending order from the centromeric end. Squares indicate males; circles indicate females; slashed symbols indicate deceased; solid symbols indicate affected; open symbols indicate unaffected; and arrow symbol indicates proband.

**Figure 2:**
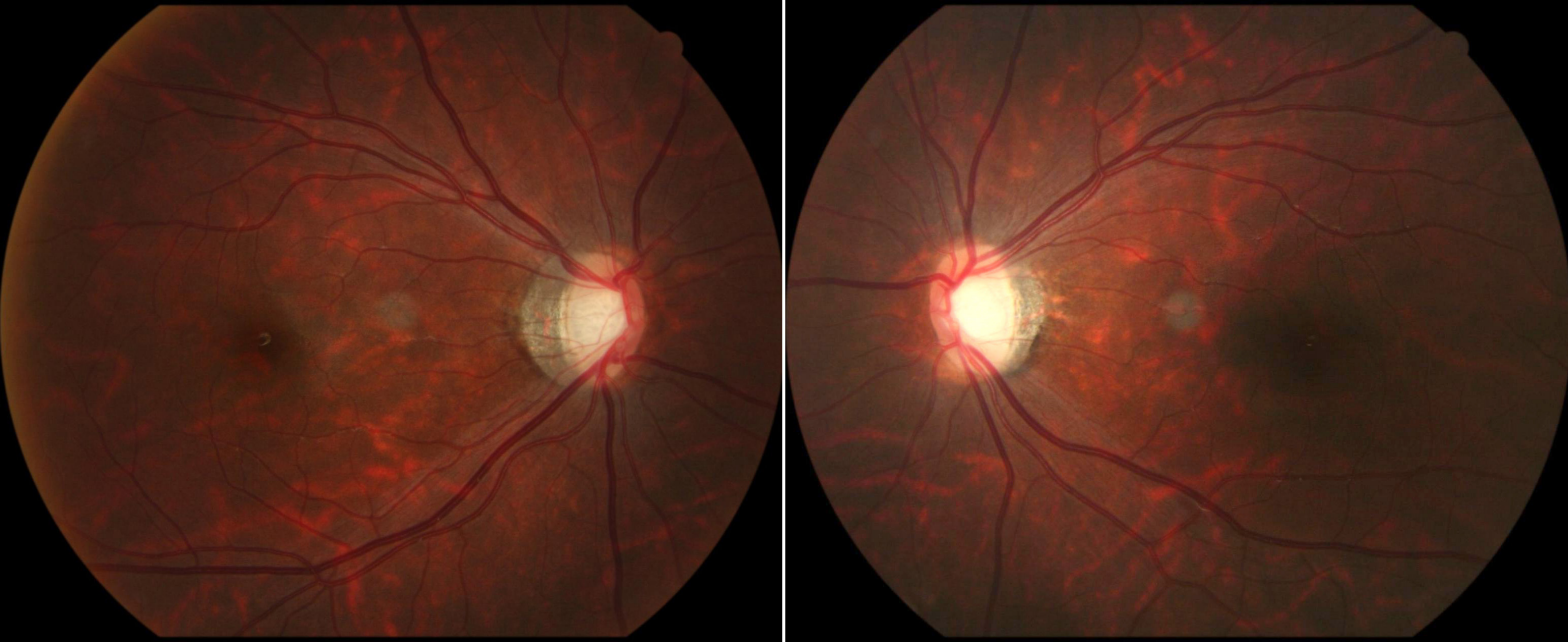
Fundus appearance of a patient with autosomal dominant optic atrophy (ADOA). Fundus of proband of family F2 showed bilateral temporal pallor of optic discs.

We have identified two unrelated Chinese families consisting of 10 patients and 9 unaffected relatives diagnosed with optic atrophy. The inheritance pattern in the two families was autosomal dominant (Figure 1). All the patients had experienced bilateral vision acuity impairment at their childhood. The ophthalmoscope demonstrated bilateral temporal or total pallor of the optic discs (Figure 2). Pseudoisochromatic plates test showed red-green color weakness. The two probands had P-VEP recordings with the decreased amplitude and prolonged latency of P100 wave. Octopus visual field of the proband in family F2 showed bilateral central scotoma. Detailed clinical information of each family’s affected member is summarized in Table 2. None of the participants from either family presented with hearing loss, cataract, ptosis, or ophthalmoplegia.

**Table 2:** Clinical features of family members in the two families

**Family number**	**Pedigree number**	**Gender**	**Age**	**Best corrected Visual acuity OD**	**Best corrected Visual acuity OS**	**Disc appearance**
F1	II:1	M	60	0.02	0.02	A
	II:2	F	56	1	1	N
	III:1	M	30	1	1	N
	III:2	M	29	1	1	N
	III:3	F	36	0.4	0.4	T
	II:4	F	53	0.02	0.02	A
	II:3	M	63	NLP*	0.8	N
	III:4	M	35	0.3	0.7	T
	III:5	F	30	1	1	N
	IV:1	F	5	0.2	0.2	T
	III:6	F	31	0.8	0.8	N
	II:6	F	51	0.04	0.02	A
	II:5	M	52	1	0.8	N
	III:7	M	20	1	1	N
	III:8	F	28	0.6	0.6	T
F2	I:1	M	46	0.1	0.1	T
	I:2	F	45	1	1	N
	II:1	M	21	0.1	0.1	T
	II:2	F	19	0.2	0.2	T

Abbreviations: male (M), female (F), total optic disc pallor (A), temporal optic disc pallor (T), normal (N), and no light perception (NLP). The asterisk indicates the eyeball had atrophied due to a corneal penetrating trauma. Clinical features of all participants from the two families with ADOA were summarized in this table.

### Genotyping results

Family F1 was genotyped with two short tandem repeat polymorphism (STRP) markers located around the *OPA1* gene in the 3q28–29 region. The marker results for D3S2305 and D3S3562 were fully informative for linkage. There was no affected recombinant for either of the 2 markers (Figure 1). Although a meiotic breakpoint was observed in an unaffected family member (III:7), the marker D3S2305 was very close to the *OPA1* locus, whereas marker D3S3562 was further telomeric and did not comprise any part of the *OPA1* gene. However, one clinical unaffected individual (III: 2) carried the affected haplotype.

### Mutation analysis

**Figure 3:**
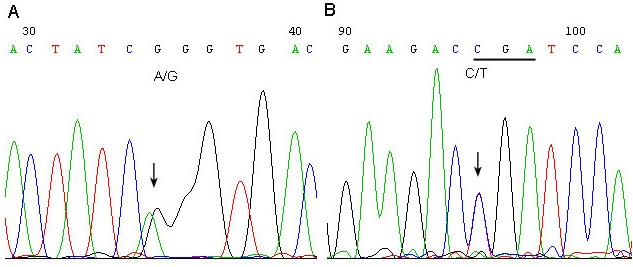
Direct sequencing analysis of the coding region of the *OPA1* gene. **A.** Sequence shows the heterozygous splicing site mutation c.985–2A>G; **B:** Sequence shows the heterozygous nonsense mutation c.2197C>T (p. R733X). The 2 sequences given are in sense direction.

After sequencing the *OPA1* gene, we identified two novel heterozygous mutations: a splicing site mutation c.985 −2A>G in family F1 and a nonsense mutation c.2197C>T (p.R733X) in family F2 (Figure 3). Using SSCP analysis, we found that these heterozygous mutations cosegregated with all affected members and the affected haplotype carrier, but we did not detect them in 100 unrelated normal controls.

**Figure 4:**
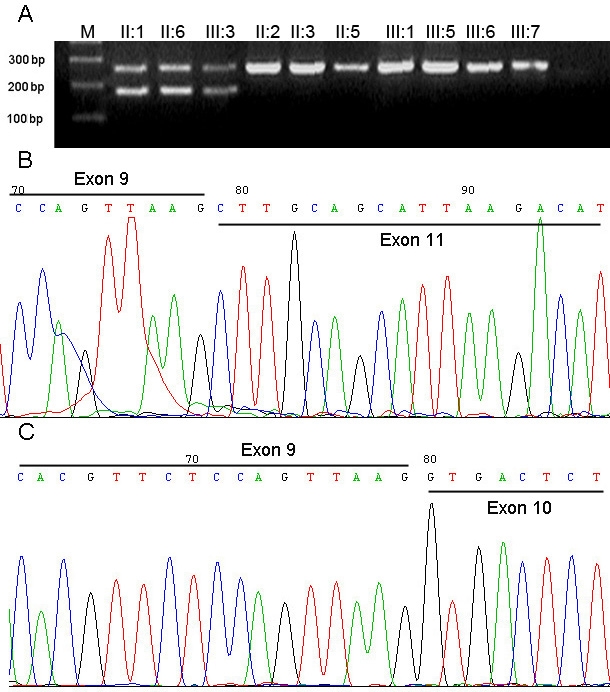
RT–PCR and direct sequencing analysis for the splice site mutation c.985–2A>G. **A:** Ethidium bromide-agarose gel was loaded with RT–PCR products generated from leukocytes of total RNA of three affected members (two bands) and seven unaffected individuals (one band) of family F1. **B** and **C:** The sequence chromatograms of the two bands from the patient’s RT–PCR products; **B:** The mutant transcript sequence with the skipping exon 10. **C:** The corresponding normal transcript sequence between exon 9 and exon 11.

To test whether the splice-site mutation lead to a defective mRNA, we performed an RT–PCR assay to amplify *OPA1* mRNA from total RNA isolated from peripheral blood of the 3 patients and 7 normal individuals. Two fragments (about 270 bp and 189 bp) were obtained from the patients, while only 1 fragment (about 270 bp) was obtained from the normal controls (Figure 4). The results of sequencing revealed that the 270 bp fragment was normal; however, the 189 bp fragment showed the skipping of exon 10 (Figure 4).

## Discussion

In this study, we examined two Chinese families with clinically diagnosed ADOA and screened the *OPA1* gene. We identified a novel heterozygous splicing site mutation c.985–2A>G and a novel heterozygous nonsense mutation c.2197C>T (p.R733X). The 2 novel mutations cosegregated with the disease phenotype and were not detected in 100 normal controls.

To date, more than 100 *OPA1* gene mutations have been reported [9–11,15–24]; however, only 2 mutations were reported in Chinese patients. The 2 mutations, a missense p.G401D and a small deletion p. D950CfsX4, are associated with a complex clinical phenotype characterized by ADOA with sensorineural deafness [21,22]. Therefore, the current study is the first report of mutations causing isolate ADOA in Chinese families. Among the mutations reported, more than 20% of them are splice site mutations, which are presumed to result in the in-frame skipping of exons or premature termination of OPA1 translation. The novel splice-site mutation c.985–2A>G detected in family F1 causes an in-frame skipping of exon 10, which encodes 27 amino acids in the highly conserved GTPase domain of OPA1 protein. Delettre et al. reported a recurrent mutation c.985–1G>A, which has a similar consequence [9,11]. This may suggest that the segment encoded by the exon 10 is critical for the GTPase activity of OPA1, and the exon 10 splice acceptor site may be a mutation hot spot.

In our review of the literature, we found ADOA displays great variability in phenotypic expression. It was originally thought that penetrance was almost complete (98%) [16]. However, the subsequent molecular analysis revealed that the disease penetrance is much lower. Revised estimates of penetrance have varied between 43% and 62% for different *OPA1* mutation [16]. Previously, only visual acuity and optic disc appearance were used to classify a mutation carrier penetrant [16]. Cohn et al. extended the clinical parameters to include color vision and visual field assessment, and observed that the overall penetrance for *OPA1* mutations in the large Australian ADOA pedigrees was 82.5% [23]. Using this clinical standard, they recalculated the penetrance of 2 families harboring c.2708del (TTAG) mutation previously reported by Toomes et al. [16]. The recalculated penetrance of the two families was 66% and 67% [23], which was higher than the original 43% and 62% [16]. In the current study, one clinically unaffected member of family F1 carried the mutation c.985–2A>G; he was also found to harbor the affected haplotype. The penetrance of ADOA for family F1 was 87.5% (7/8). The limitation of the study was that only visual acuity and optic disc appearances were used to classify the mutation carrier penetrant.

The novel nonsense mutation c.2197C>T (p.R733X) detected in family F2 presumably caused premature termination of the OPA1 translation. The predicted effect of this mutation would be a truncated protein lacking the 227 amino acids, accounting for loss of 26.6% of OPA1, including the part of central dynamin domain and total coiled-coil region at the C-terminus. As the premature stop codons are not located in the last exon of the *OPA1* gene, there is a possibility that nonsense-mediated mRNA decay (NMD) might be involved in the abnormal RNAs processing. NMD is a widespread cellular process that proofreads nascent mRNA transcripts and destroys those that bear premature termination codons before they are actually translated in truncated and potentially harmful proteins [26]. In a recent study, Schimpf et al. demonstrated that the majority of nonsense *OPA1* mutations underwent NMD. Using pyrosequencing of an RT–PCR amplified cSNP (c.2109C>T) in *OPA1*, they found that mutant transcript levels reduced between 1.25- and 2.5-fold and varied among premature termination codons containing mutations [24].

In conclusion, we described 2 novel mutations of the *OPA1* gene in Chinese ADOA families. Our findings expand the spectrum of *OPA1* gene mutation and provided useful genetic consultation and genetic diagnosis for the families.

## References

[1] Kjer B, Eiberg H, Kjer P, Rosenberg T (1996). Dominant optic atrophy mapped to chromosome 3q region. II. Clinical and epidemiological aspects.. Acta Ophthalmol Scand.

[2] Lyle WM. Genetic risks: a reference for eye care practitioners. Waterloo (Canada): University of Waterloo Press; 1990.

[3] Votruba M, Moore AT, Bhattacharya SS (1998). Clinical features, molecular genetics, and pathophysiology of dominant optic atrophy.. J Med Genet.

[4] Johnston PB, Gaster RN, Smith VC, Tripathi R (1979). A clinicopathologic study of autosomal dominant optic atrophy.. Am J Ophthalmol.

[5] Eiberg H, Kjer B, Kjer P, Rosenberg T (1994). Dominant optic atrophy (OPA1) mapped to chromosome 3q region I. Linkage analysis.. Hum Mol Genet.

[6] Kerrison JB, Arnould VJ, Ferraz Sallum JM, Vagefi MR, Barmada MM, Li Y, Zhu D, Maumenee IH (1999). Genetic heterogeneity of dominant optic atrophy, Kjer type: identification of a second locus on chromosome 18q12.2–12.3.. Arch Ophthalmol.

[7] Reynier P, Amati-Bonneau P, Verny C, Olichon A, Simard G, Guichet A, Bonnemains C, Malecaze F, Malinge MC, Pelletier JB, Calvas P, Dollfus H, Belenguer P, Malthièry Y, Lenaers G, Bonneau D (2004). OPA3 gene mutations responsible for autosomal dominant optic atrophy and cataract.. J Med Genet.

[8] Barbet F, Hakiki S, Orssaud C, Gerber S, Perrault I, Hanein S, Ducroq D, Dufier JL, Munnich A, Kaplan J, Rozet JM (2005). A third locus for dominant optic atrophy on chromosome 22q.. J Med Genet.

[9] Delettre C, Lenaers G, Griffoin JM, Gigarel N, Lorenzo C, Belenguer P, Pelloquin L, Grosgeorge J, Turc-Carel C, Perret E, Astarie-Dequeker C, Lasquellec L, Arnaud B, Ducommun B, Kaplan J, Hamel CP (2000). Nuclear gene *OPA1*, encoding a mitochondrial dynamin-related protein, is mutated in dominant optic atrophy.. Nat Genet.

[10] Alexander C, Votruba M, Pesch UE, Thiselton DL, Mayer S, Moore A, Rodriguez M, Kellner U, Leo-Kottler B, Auburger G, Bhattacharya SS, Wissinger B (2000). *OPA1*, encoding a dynamin-related GTPase, is mutated in autosomal dominant optic atrophy linked to chromosome 3q28.. Nat Genet.

[11] Delettre C, Griffoin JM, Kaplan J, Dollfus H, Lorenz B, Faivre L, Lenaers G, Belenguer P, Hamel P (2001). Mutation spectrum and splicing variants in the *OPA1* gene.. Hum Genet.

[12] Olichon A, Emorine LJ, Descoins E, Pelloquin L, Brichese L, Gas N, Guillou E, Delettre C, Valette A, Hamel CP, Ducommun B, Lenaers G, Belenguer P (2002). The human dynamin-related protein OPA1 is anchored to the mitochondrial inner membrane facing the inter-membrane space.. FEBS Lett.

[13] Satoh M, Hamamoto T, Seo N, Kagawa Y, Endo H (2003). Differential sublocalization of the dynamin-related protein OPA1 isoforms in mitochondria.. Biochem Biophys Res Commun.

[14] Olichon A, Baricault L, Gas N, Guillou E, Valette A, Belenguer P, Lenaers G (2003). Loss of OPA1 perturbates the mitochondrial inner membrane structure and integrity, leading to cytochrome c release and apoptosis.. J Biol Chem.

[15] Han J, Thompson-Lowrey AJ, Reiss A, Mayorov V, Jia H, Biousse V, Newman NJ, Brown MD (2006). OPA1 mutations and mitochondrial DNA haplotypes in autosomal dominant optic atrophy.. Genet Med.

[16] Toomes C, Marchbank NJ, Mackey DA, Craig JE, Newbury-Ecob RA, Bennett CP, Vize CJ, Desai SP, Black GCM, Patel N, Teimory M, Markham AF, Inglehearn CF, Churchill AJ (2001). Spectrum, frequency and penetrance of *OPA1* mutations in dominant optic atrophy.. Hum Mol Genet.

[17] Pesch UE, Leo-Kottler B, Mayer S, Jurklies B, Kellner U, Apfelstedt-Sylla E, Zrenner E, Alexander C, Wissinger B (2001). *OPA1* mutations in patients with autosomal dominant optic atrophy and evidence for semi-dominant inheritance.. Hum Mol Genet.

[18] Thiselton DL, Alexander C, Taanman JW, Brooks S, Rosenberg T, Eiberg H, Andreasson S, Regemorter NV, Munier FL, Moore AT, Bhattacharya SS, Votruba M (2002). A comprehensive survey of mutations in the *OPA1* gene in patients with autosomal dominant optic atrophy.. Invest Ophthalmol Vis Sci.

[19] Baris O, Delettre C, Amati-Bonneau P, Surget MO, Charlin JF, Catier A, Derieux L, Guyomard JL, Dollfus H, Jonveaux P, Ayuso C, Maumenee I, Lorenz B, Mohammed S, Tourmen Y, Bonneau D, Malthiery Y, Hamel C, Reynier P (2003). Fourteen novel OPA1 mutations in autosomal dominant optic atrophy including two de novo mutations in sporadic optic atrophy.. Hum Mutat.

[20] Ferré M, Amati-Bonneau P, Tourmen Y, Malthièry Y, Reynier P (2005). eOPA1: An Online Database for OPA1 Mutations.. Hum Mutat.

[21] Ke T, Nie SW, Yang QB, Liu JP, Zhou LN, Ren X, Liu JY, Wang Q, Liu MG (2006). The G401D mutation of OPA1 causes autosomal dominant optic atrophy and hearing loss in a Chinese family.. Zhonghua Yi Xue Yi Chuan Xue Za Zhi.

[22] Chen S, Zhang Y, Wang Y, Li W, Huang S, Chu X, Wang L, Zhang M, Liu Z (2007). A novel *OPA1*mutation responsible for autosomal dominant optic atrophy with high frequency hearing loss in a Chinese family.. Am J Ophthalmol.

[23] Cohn AC, Toomes C, Potter C, Towns KV, Hewitt AW, Inglehearn CF, Craig JE, Mackey DA (2007). Autosomal dominant optic atrophy: Penetrance and expressivity in patients with *OPA1* mutations.. Am J Ophthalmol.

[24] Schimpf S, Fuhrman N, Schaich S, Wissinger B (2008). Comprehensive cDNA study and quantitative transcript analysis of mutant *OPA1* transcripts containing premature termination codons.. Hum Mutat.

[25] Bassam BJ, Caetano-Anollés G, Gresshoff PM (1991). Fast and sensitive silver staining of DNA in polyacrylamide gels.. Anal Biochem.

[26] Hentze MW, Kulozik AE (1999). A perfect message: RNA surveillance and nonsense-mediated decay.. Cell.

